# Natural history of 39 patients with Achondroplasia

**DOI:** 10.6061/clinics/2018/e324

**Published:** 2018-06-19

**Authors:** Jose Ricardo Magliocco Ceroni, Diogo Cordeiro de Queiroz Soares, Larissa de Cássia Testai, Rachel Sayuri Honjo Kawahira, Guilherme Lopes Yamamoto, Sofia Mizuho Miura Sugayama, Luiz Antonio Nunes de Oliveira, Debora Romeo Bertola, Chong Ae Kim

**Affiliations:** I Unidade de Genetica, Instituto da Crianca (ICR), Hospital das Clinicas HCFMUSP, Faculdade de Medicina, Universidade de Sao Paulo, Sao Paulo, SP, BR; II Centro de Pesquisas sobre o Genoma Humano e Celulas-Tronco (CEGH-CEL), Instituto de Biociencias (IB), Universidade de Sao Paulo, Sao Paulo, SP, BR; III Unidade de Radiologia, Instituto da Crianca (ICR), Hospital das Clinicas HCFMUSP, Faculdade de Medicina, Universidade de Sao Paulo, Sao Paulo, SP, BR

**Keywords:** Achondroplasia, Natural History, Growth, *FGFR3*

## Abstract

**OBJECTIVES:**

To characterize the natural history of 39 achondroplastic patients diagnosed by clinical, radiological and molecular assessments.

**METHODS:**

Observational and retrospective study of 39 patients who were attended at a public tertiary level hospital between 1995 and 2016.

**RESULTS:**

Diagnosis was made prenatally in 11 patients, at birth in 9 patients and within the first year of life in 13 patients. The most prevalent clinical findings were short stature, high forehead, trident hands, genu varum and macrocephaly. The most prevalent radiographic findings were rhizomelic shortening of the long bones and narrowing of the interpediculate distance of the caudal spine. There was motor developmental delay in 18 patients and speech delay in 16 patients. The most common clinical intercurrences were middle ear dysfunction, sleep apnea, limb pain and obesity from 2 to 9 years of age. One patient was large for the gestational age but did not develop obesity. One patient developed hydrocephalus at 10 years old. The current age of the patients varies from 15 months to 36 years. The molecular study performed by Sanger sequencing of the common heterozygous mutation 1138G>A in *FGFR3* was positive in all patients. Four cases were inherited, and 35 were sporadic (paternal age from 19 to 66 years).

**CONCLUSIONS:**

The diagnoses were made early based on clinical and radiographic findings. All cases were confirmed molecularly. Despite presenting a benign course, it is necessary to establish a systematic protocol for the surveillance of these patients due to the common clinical intercurrences.

## INTRODUCTION

Achondroplasia (OMIM #10800) is the most common non-lethal chondrodysplasia with an estimated incidence of 2.6:100,000. It is caused by missense mutations in *FGFR3* (fibroblast growth factor receptor 3), with an autosomal dominant pattern of inheritance and complete penetrance. Most cases are *de novo*. In general, diagnosis is possible through clinical and radiological findings recognizable at birth. Clinical findings include disproportionately short stature, characteristic faces with frontal bossing and midfacial retrusion, brachydactyly, a trident appearance of the hands, lumbar hyperlordosis, genu varum and joint hypermobility. The main radiological findings are rhizomelic shortening of the long bones and narrowing of the interpediculate distance of the caudal spine. In cases in which there is doubt concerning the clinical diagnosis, molecular testing is advised. Most patients have good health with a benign course of the condition, and there is no consensus regarding a clinical protocol for follow-up. The main medical intercurrences related to the disease are delayed motor milestones, obesity, hydrocephalus, craniocervical junction compression, obstructive sleep apnea, otorhinolaryngologic diseases, spinal stenosis, deformity of limbs, difficulty of socialization and depression.

The objective of this work is to characterize the natural history of 39 achondroplastic patients diagnosed by clinical, radiological, and molecular investigations.

## MATERIALS AND METHODS

We performed an observational and retrospective study of 43 patients with molecularly confirmed diagnoses of achondroplasia from 1995 to 2016. Four patients were excluded from the study due to being lost to follow-up. An additional 3 patients had clinical and radiological evidence, but are not included in this study due to the lack of molecular studies. The included patients are attended at the skeletal dysplasia medical ambulatory clinic, which provides regular follow-up for a total of 150 patients with different forms of nanism.

The study was approved by the research ethics commission for the analysis of research projects (CAPPesq) of the Hospital das Clinicas Medical School of the University of São Paulo (HC-FMUSP).

Clinical follow-up was performed annually when possible and consisted of the assessment of prenatal and birth information obtained from records; physical examination of height, weight, head circumference measurements, and neurologic and dysmorphic evaluations; the assessment of developmental milestones; and a review of the family history.

Radiologic studies consisted of complete body X-rays of all patients.

The molecular study consisted of Sanger sequencing of exon 9 of *FGFR3.* (NM_000142)

## RESULTS

### Etiology

In our patients, sporadic cases were the majority – 36 out of 40 (90%). Four patients were familial cases, with two affected mothers and one affected father. The maternal age varied from 19 to 39 years (mean 30.6 years and median 31 years). The paternal age at conception of the sporadic cases varied from 19 to 66 years (mean 37.2 years and median 34 years).

### Diagnosis

The diagnosis was made in the prenatal period by ultrasound with findings of shortening of the long bones in 16/39 (41%) patients. In nine (23%) patients, the diagnosis was made at birth, and in 13 (33%) patients, the diagnosis was made within the first year. The latest diagnosis was made at 4 years old. The current age of our 39 patients varies from 15 months to 36 years (mean of 10.2 years and median of 6.5 years).

### Neonatal Findings

Cesarean section was performed in 29/39 (74.3%) patients. Thirty-four patients were born at term, 4/39 (10.2%) were premature and 1/39 (2.6%) was born post-term.

Birth weight varied from 2170 g to 4700 g (mean 3165 g and median 3235 g). Low birth weight (<2500 g) was present in 4/39 (10.2%) patients, three who were premature and one whose mother had hypertensive disease during pregnancy. Birth length varied from 42 cm to 53 cm (mean 46 cm and median 45.7 cm). Birth head circumference varied from 33 cm to 45 cm (mean 36.6 cm and median 36.5 cm).

Perinatal intercurrence occurred in 9/39 (23%) patients. Three patients were premature, presenting respiratory distress, pathological jaundice and deglutition disturbance. One patient required three months of hospitalization. Other common findings were perinatal asphyxia and sepsis.

### Clinical Findings

**Figure 1:**
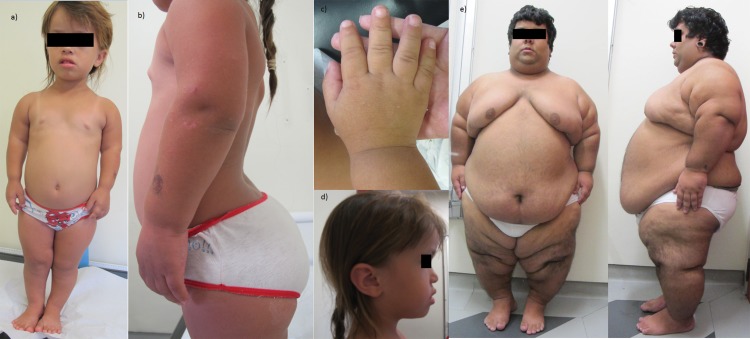
Clinical findings in two achondroplastic patients. a-c) The clinical findings in a 6-year-old female patient. a) Anterior view of the patient shows rhizomelic short-limb dwarfism and macrocephaly; b) the profile view demonstrates the common prominent kyphosis and lumbar lordosis; c) the typical hand in this patient shows brachydactyly with a trident configuration; d) the profile picture demonstrates the common phenotypic changes in achondroplasia: frontal bossing, midface hypoplasia and low nasal bridge; e) a 20-year-old male patient whose weight is 148 kg and whose height is 139.5 cm.

The main clinical findings were: dwarfism in 39/39 (100%) patients, frontal bossing in 38/39 (97%) patients, trident hands in 38/39 (97%) patients, genu varum in 38/39 (97%) patients, macrocephaly in 33/39 (84.6%) patients, thoracolumbar kyphosis in 27/39 (69%) patients and joint hypermobility in 13/39 (33%) patients (Figure 1).

### Clinical Intercurrence

The most common intercurrences were middle ear dysfunction, sleep apnea, obesity and orthopedic abnormalities (pain and excessive bowing of the lower limbs).

Recurrent infection of the middle ear occurred in 13/35 (37%) patients, persistent middle ear fluid in 3/35 (9%), hearing loss in 4/35 (11%), and ventilation tube insertion was performed for 4/35 (11%) patients.

Sleep disturbance was reported in 21/39 (53.8%) of patients from the first month to 10 years of life (mean 3.94 years and median 3 years old). Polysomnography was performed in 11 patients, and all showed sleep apnea. Adenotonsillectomy was performed in 5 patients, and one patient underwent the procedure twice.

Obesity was observed in 4/39 (10%) patients and occurred from 2 years to 9 years (mean and median of 6 years). One patient at 20 years old weighed 148 kg with a height of 139.5 cm (BMI 76.1 kg/m^2^); this patient also suffered from depression, excessive snoring, sleep apnea and dyspnea at rest and was referred for multidisciplinary care.

Pain in lower limbs occurred in 10/39 (25.6%) patients, and excessive bowing of limbs in 4/39 (10.3%).

In terms of developmental milestones, head control was achieved from 2 to 12 months of age (mean and median of 6 months), sitting independently was achieved from 5 to 20 months (mean 9.3 months and median 8 months), and walking independently was achieved from 7 to 50 months (mean 18.6 months and median 17 months). Language developmental delay was observed in 16/39 (41%) patients.

All patients attend regular school with good performance.

One patient developed hydrocephalus at 10 years old. Other complications related to the constriction of the craniocervical junction and spinal cord stenosis were not reported. One patient developed unrelated epilepsy.

One patient developed bilateral Wilms’ tumor at three years old, for which chemotherapy and unilateral nephrectomy were performed.

### Radiological findings

**Figure 2:**
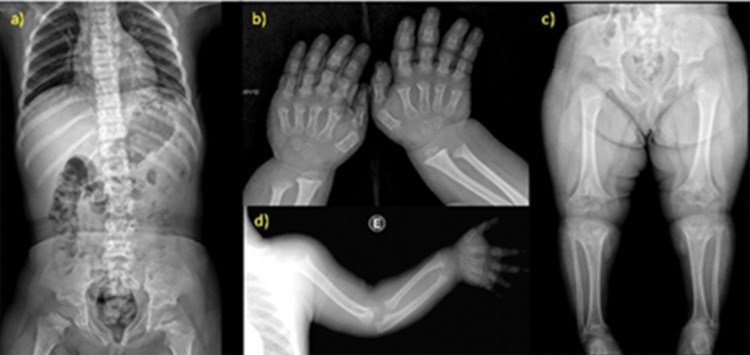
Radiological findings of achondroplasia. a-d) X-ray studies of a 5-year-old patient. a) Anterior view of the vertebral column shows narrowing of the interpediculate distance of the caudal spine; b) the typical hand presents brachydactyly with a trident configuration; c) the pelvis has rounded ilia and horizonal acetabula, and the femurs are short with proximal radiolucency; d) the short humerus also demonstrates rhizomelic dwarfism.

The most common radiological findings were rhizomelic shortening of the long bones in 39/39 (100%) patients; narrowing of the interpediculate distance of the caudal spine in 34/39 (87%) patients; rounded ilia and horizontal acetabula in 32/39 (82%) patients; mild, generalized metaphyseal changes in 30/39 (73%) patients; proximal femoral radiolucency in 27/39 (70%) patients; and narrow sacrosciatic notch in 24/39 (62%) patients. These findings are compiled in Figure 2.

Cranial CT scans were done in 15/16 (94% patients (mean 3.4 years and median 1 year of age), and all patients except one presented reduced foramen magnum size.

### Molecular study

The molecular study consisted of Sanger sequencing of exon 9 of *FGFR3* (NM_000142). The common heterozygous mutation 1138G>A was detected in all patients.

## DISCUSSION

### Diagnosis

Achondroplasia is a condition that is easily recognizable in clinical and radiological findings. Early recognition of the syndrome is important because it supports health providers’ decisions concerning type of labor, peripartum care, and prognosis, as well as decreasing familial anxiety.

Ultrasonographic changes compatible with achondroplasia are observable by 20 weeks of gestation and include the rhizomelic shortening of limbs, a large head with frontal bossing, midfacial retrusion and depressed nasal bridge, and a trident configuration of the hands. The thoracoabdominal ratio is invariably >0.7, which is important information at evaluation because of the other differential diagnoses, including thanatophoric dysplasia, in which this ratio is frequently <0.7, indicating a poor postnatal prognosis due to a very narrow thorax and respiratory difficulties.

In our study, the diagnosis was made at an early stage. By the first year of life, a total of 33 (89%) already had clinical diagnoses.

### Clinical findings

The most prevalent clinical findings were small statures with rhizomelic shortening of limbs, large heads with frontal bossing, midfacial retrusion and depressed nasal bridges, trident configuration of the hands, and genu varum. These findings are almost invariable in this condition and were also present in our patients.

Neonatal intercurrences are not expected in patients with achondroplasia unless other risk factors not related to the disease are present, such as prematurity or hypertensive disorder of pregnancy. It is important to note that achondroplasic mothers must undergo caesarian section due to the small size of the pelvis.

At birth, the weight of the patients was normal, length was somewhat compromised, and the head circumference was larger.

Anthropometric measures at birth of our patients were in accordance with those expected for the disease, except for one patient who was born large for the gestational age (40 w and 4700 g). This finding is not related to the disease and was probably due to maternal causes.

In general, by six months of age, the length was below -2 standard deviations (SD), and the head circumference was above +1SD. It is noteworthy that there are specific growth charts 1 for achondroplasic patients and recommendations for regular anthropometric evaluation 2.

### Medical complications

The most prevalent clinical intercurrences of patients with achondroplasia are middle ear dysfunction, sleep apnea, obesity, craniocervical junction constriction, spinal stenosis, hydrocephalus, limb deformity and pain, kyphosis, difficulty in socialization and depression 2,3.

Short eustachian tubes may lead to recurrent infections of the middle ear with persistent middle ear fluid; therefore, ventilation tube insertion may be indicated 4 to prevent hearing loss. The cause of upper airway obstruction in achondroplasia is multifactorial. There are anatomic causes that lead to obstruction predisposing the patient to sleep apnea, which can also be aggravated by adenoid hypertrophy. Patients who undergo adenotonsillectomy are at risk of respiratory intercurrences in post-operatory care and require careful monitoring in the intensive care unit 4. Obesity may be difficult to manage due to the short stature of the patients, and it also aggravates other health problems such as sleep apnea, spine and articulation pain, and deformity of limbs. It is recommended that patients are stimulated by the medical team and the family to consume a balanced diet and to practice exercise. Despite these recommendations, it is important to inform the families that the patients must avoid some activities, due to the risk of injury to the spinal at the craniocervical junction, such as collision sports, trampoline use, diving from boards, vaulting in gymnastics, and hanging upside down from knees or feet on the playground.

Most patients are referred for orthopedic care and for surgical evaluation due to the common findings of limb pain, and limb deformity.

These findings were also prevalent in our patients. Sleep apnea was present in 21/39 (54%) patients, middle ear dysfunction in 19/35 (54%) patients, limb pain in 10/39 (26%) patients, and obesity in 4/39 (10%) patients. In our cases, obesity occurred in four patients, with early onset (from 2y to 9y). One patient had morbid obesity (148 kg and 139 cm) with severe respiratory complications and was referred for multidisciplinary care with a good response.

Approximately 3-7% of patients with achondroplasia die of sudden death in the first year of life due to the compression of the brainstem or obstructive sleep apnea 5. All our patients are alive.

Two patients developed unexpected intercurrences: one patient developed epilepsy, and the other presented at 5 years of age with bilateral Wilms’ tumor and underwent a partial nephrectomy of one kidney and the total nephrectomy of the other.

### Developmental milestones

Motor development is constantly delayed in achondroplasic patients due to the large head size compared to the small body and the transient hypotonia 6,7. There are specific motor development charts for patients with achondroplasia 8, which state that head control is expected to be achieved at 3-9 months of age, sitting independently to be achieved at 7 to 14 months of age, and walking independently to be achieved at 15 to 30 months of age.

In our study, 18 (46%) patients presented with motor developmental delay.

The first words in patients with achondroplasia are expected to be spoken at 7 to 17 months 8. Speech delay, when present, is not related to the disease and is probably due to the lack of external stimulation we hypothesize in our patients. Intellectual impairment does not occur in the disease, and patients can live productively.

In contrast to the results in the literature, our study showed 16 (41%) patients with speech delay. All patients attend regular school with good performance.

### Image findings

X-ray findings are rhizomelic shortening of long bones, which also become robust and tubular bones, a narrowing of the interpediculate distance of the caudal spine, rounded ilia and horizontal acetabula, a narrow sacrosciatic notch, proximal femoral radiolucency, and mild, generalized metaphyseal changes 9. Virtually all patients with achondroplasia have reduced foramen magnum size as determined by cranial CT examination. Reduced foramen magnum size might cause compression of the brainstem at the craniocervical junction and therefore results in elevated frequency of hypotonia, tetraparesia, motor developmental delay, central apnea, and sudden death 5.

The most prevalent radiographic findings of our patients were in accordance with those in the literature; we found rhizomelic shortening of long bones in 39 (100%) patients and narrowing of the interpediculate distance of the caudal spine in 34 (87%) patients.

### Molecular study

Achondroplasia is a monogenic disorder caused by two specific mutations in *FGFR3* that are responsible for more than 99% of the cases: 1138G>A in approximately 97% and 1138G>C in approximately 2% of the cases 10. Both mutations result in the substitution Gli380Arg. The p.Gly380Arg mutation causes a constitutive activation of *FGFR3*, which is a negative regulator of bone growth 11. These changes result in excess inhibitory signaling in growth plate chondrocytes 12,13.

Achondroplasia cases are due to *de novo* mutations in 80% of cases. *De novo* mutations in guanine at the 1138 position of *FGFR3* occur mostly in the paternal germline and increase in frequency with high paternal age (above 35 years) 14,15,16.

All our patients presented the classical mutation 1138G>A in heterozygosity in *FGFR3*. Among 39 patients, 35 were *de novo* sporadic cases and four were familial. Paternal age varied in sporadic cases from 19 to 66 years (mean 38 and median 34 years) and 15/35 (43%) of these fathers were above 35 years old at conception.

Mutations in *FGFR3* cause other disorders such as hypochondroplasia, thanatophoric dysplasia and SADDAN (severe achondroplasia with developmental delay and acanthosis nigricans). In hypochondroplasia, the skeletal features are similar but milder than those seen in achondroplasia. Medical complications (spinal stenosis, tibial bowing, obstructive apnea) occur less frequently, but intellectual disability and epilepsy are more prevalent. Thanatophoric dysplasia (TD) is a lethal dwarfism syndrome that presents with cloverleaf skull, short ribs, narrow thorax, brachydactyly, and evident skin folds along the limbs due to severe micromelia. SADDAN has a similar phenotype to TD, with the hallmark of acanthosis nigricans.

In summary, achondroplasia is usually considered a benign disorder, and medical complications are often neglected. There are not yet well-established protocols for surveillance. Most of existing protocols are made based on the medical center’s experience or based on the patients’ individual needs 17.

## AUTHOR CONTRIBUTIONS

Ceroni JR was responsible for the collection of data and manuscript writing. Soares DC performed technical corrections of the manuscript. Testai LC performed Sanger sequencing and analysis. Kawahira RS was responsible for technical and bibliographic corrections. Yamamoto GL was responsible for technical corrections and X-ray analysis. Sugayama SM was responsible for technical and bibliographic corrections. Oliveira LA was responsible for X-ray and CT analysis. Bertola DR was responsible for the clinical management of the patients. Kim CA proposed the idea of the manuscript and was responsible for the clinical management of the patients.
